# Depression Screening and Education: Options to Reduce Barriers to Treatment (DESEO): protocol for an educational intervention study

**DOI:** 10.1186/s12913-016-1575-3

**Published:** 2016-07-29

**Authors:** Katherine Sanchez, Brittany H. Eghaneyan, Madhukar H. Trivedi

**Affiliations:** 1 School of Social Work, University of Texas at Arlington, 211 South Cooper Street, Arlington, TX 76019 USA; 2 Department of Psychiatry, UT Southwestern Medical Center, 5323 Harry Hines Blvd., Dallas, TX 75390-9119 USA

**Keywords:** Depression, Education, Hispanics, Stigma, Primary care, iPad screening, *Fotonovela*

## Abstract

**Background:**

Barriers to depression treatment among Hispanic populations include persistent stigma, inadequate doctor patient communication (DPC) and resultant sub-optimal use of anti-depressant medications. Stigma is primarily perpetuated due to inadequate disease literacy and cultural factors. Common concerns about depression treatments among Hispanics include fears about the addictive and harmful properties of antidepressants, worries about taking too many pills, and the stigma attached to taking psychotropic medications. The current manuscript presents the study protocol for the Depression Screening and Education: Options to Reduce Barriers to Treatment (DESEO) study funded by the Center for Medicare and Medicaid Services (CMS) Grants to Support the Hispanic Health Services Research Grant Program.

**Methods/Design:**

DESEO will implement universal screening with a self-report depression screening tool (the 9-item Patient Health Questionnaire (PHQ-9)) that is presented through a customized web application and a Depression Education Intervention (DEI) designed to increase disease literacy, and dispel myths about depression and its treatment among Hispanic patients thus reducing stigma and increasing treatment engagement. This project will be conducted at one community health center whose patient population is majority Hispanic. The target enrollment for recruitment is 350 patients over the 24-month study period. A one-group, pretest-posttest design will be used to asses knowledge of depression and its treatment and related stigma before, immediately after, and one month post intervention.

**Discussion:**

Primary care settings often are the gateway to identifying undiagnosed mental health disorders, particularly for people with comorbid physical health conditions. This study is unique in that it aims to examine the specific role of patient education as an intervention to increase engagement in depression treatment. By participating in the DEI, it is expected that patients will have time to understand treatment options, participate in shared decision-making with their provider, and increase engagement in treatment of depression which might lead to improved overall health. It is also expected that implementation of the iPad Depression Screening application will increase provider awareness of the incidence and prevalence of depression in their own practice and improve the performance and care the clinic provides.

**Trial registration:**

The study was registered with: NCT02491034 July 2, 2015.

## Background

### Hispanics and depression

Clinical depression is the leading cause of medical disability, health burden, and increased medical cost in the United States and the world, costing an estimated $ 83–125 billion in the U.S. each year, and more than half these expenses are publicly funded [1]. It is estimated that the lifetime prevalence of a psychiatric disorder for Hispanics residing in the United States is 28.1 % for men and 30.2 % for women. Hispanics are more likely to experience a psychiatric disorder if they are born in the United States, are proficient in the English language, or are third-generation in the United States [2]. Hispanics have a higher prevalence of diabetes but also have double the risk of comorbid depression than the general population, with rates as high as 33 % [3].

For many Hispanics, response to depression treatment may require a considerable amount of time to reach remission, as much as two and a half years [4] Additionally, relapse rates are high and the slow response to treatment may explain the premature discontinuation of medication by patients. Intractable symptoms and slow recovery leave minority populations with a considerable disease burden for a substantial length of time compared to non-Latino whites [4, 5].

Low use of anti-depressant medication, poor doctor patient communication, and persistent stigma around issues of mental illness are key barriers to the treatment of depression in Hispanic populations [4, 6, 7]. Hispanics initiate anti-depressant medication treatment at a much lower rate than whites, and are more likely to discontinue their treatment for depression without consulting their physician, in spite of being equally likely as whites to have received a medication prescription from their primary care provider [8]. Common concerns about depression treatments include fears about the addictive and harmful properties of antidepressants, worries about taking too many pills, and the stigma attached to taking psychotropic medications [9, 10]. Somatic presentation of depressive symptoms, especially in the context of comorbid illness, impedes accurate and timely detection by primary care providers and, in settings where systematic screening for depression is absent, barriers to treatment are further amplified [11].

### Primary care

By 2003, 54 % of people with mental health disorders were served in primary care, without referral to specialty mental health [12], supporting the description of primary care as the ‘de facto’ mental health care system [13]. Hispanics, in particular, are more likely to receive mental health care in primary care settings [14]. Many reasons have been cited for this trend, from lack of access to mental health specialists, income and insurance barriers, to the trust of the relationship with the family physician [15, 16]. Additionally, low English proficiency is associated with reports of poor quality of primary care, an absence of a source of care, and a lack of continuity [17]. Other studies conclude that treatment and linguistic barriers are likely to be even more pronounced at the community level for Hispanics [18–21].

### Importance of screening

Self-report depression screening instruments can save provider time, minimize stigma, and engage patients in the identification of their symptoms of depression. Screening instruments can be powerful tools in assisting primary care providers with detecting depression in their patient population, diagnosing depression and monitoring treatment response. These instruments can also help track a patient’s overall depression severity as well as the specific symptoms that are improving or not with treatment [22]. The most recent recommendations from the U.S. Preventive Services Task Force are that primary care providers screen adult patients for depression only if systems are in place to ensure treatment and follow-up [23].

### Role of education

Management of depression among Hispanics in community-based safety net settings is confounded by issues of disease literacy, cultural treatment preferences and financial barriers to care [24, 25]. The role of patient education in management of chronic disease to increase patient engagement and improve health outcomes has been well established [26]. However, less is known about patient education and its role in mental illness [27]. Depression education interventions, which focus on symptom recognition, stress reduction techniques, and behavioral activation to reduce barriers to treatment, show promise [28, 29].

Educational interventions to proactively address barriers to depression treatment for Hispanic patients have included adapting materials for literacy and cultural content, reimbursing patients for completing outcome measures, and covering transportation expenses. Additional strategies include offering the patients the opportunity to include family members in sessions, if preferred, with a particular focus on linking patient education to depression self-management and socioeconomic stressors created by their illness. Provision of patient education and homework materials that are linguistically, idiomatically, and literacy-level appropriate is key [30].

### Aims

The current paper provides a description of the design of DESEO: Depression Screening and Education: Options to Reduce Barriers to Treatment. The primary aim of DESEO is to implement a Depression Education Intervention (DEI) designed to increase disease literacy, and dispel myths about depression and its treatment among Hispanic patients thus reducing stigma and increasing treatment engagement. The secondary aim of the study is to test the feasibility of universal screening for depression utilizing the 9-item Patient Health Questionnaire (PHQ-9) [31] via the iPad Depression Screening application.

### Objectives

1. To increase knowledge of depression (signs/symptoms, causes, risk factors, treatment, cultural beliefs, and its role in chronic disease) among Hispanics in a primary care setting.
2. To reduce perceived cultural stigma about depression and its treatment through a culturally and linguistically appropriate educational intervention.
3. To increase engagement in depression treatment in primary care by Hispanic patients.
4. To systematically screen all adult primary care patients with an iPad Depression Screening application.
5. To increase provider detection of depression in patients through the proper use and interpretation of the PHQ-9.

### Hypotheses

Our hypothesis is that the provision of depression education to Hispanic patients in a community based health center will lead to an increase in the understanding of depression, a reduction in stigma and an increase in patient engagement in treatment. Additionally, we hypothesize that the implementation of the iPad Depression Screening application will increase provider awareness of the incidence and prevalence of depression in their own practice and thus improve the performance and care the clinic provides.

## Methods/Design

### Setting and participants

The study will take place at one Federally Qualified Health Center that primarily serves a Hispanic population in north Texas and whose mission is to provide a community medical home through accessible, compassionate and quality health care services. The center offers a full range of quality family-oriented comprehensive primary and preventive services, including Family Medicine, Pediatrics, Obstetrics/Gynecology, family planning services, prenatal care, and health education promotion and disease prevention services. In 2013, the clinic had 11,274 unduplicated patients and 25,362 patient visits. Of those patients, 83 % were Hispanic. The majority did not speak English.

The target enrollment for recruitment is 350 patients over the 24-month study period. All adult primary care patients at the clinic will be asked to participate in screening for depression with the iPad Depression Screening application (universal screening). The clinic sees an average of 450–500 unique adult patients per month (new and/or unduplicated) or 100–125 patients per week. It is expected that *at least* 10 % of the patient population will screen positive for depression. Patients that are not Hispanic will be ineligible for the study because the intervention is culturally and linguistically adapted for a Hispanic population. Patients who are already in treatment for depression (medication and/or psychotherapy) will also be excluded since treatment engagement is an expected outcome. The study protocol was reviewed and approved by the Institutional Review Board of the University of Texas at Arlington. All patients will provide written informed consent to participate.

### Study design and intervention

This project will implement universal screening for depression and a Depression Education Intervention (DEI) to increase detection, diagnosis, and treatment of depression at one community health center whose patient population is majority Hispanic. The project will begin with a self-report depression screening tool that is administered to all clinic patients (universal screening) through a customized web application using an iPad. This iPad Depression Screening application technology represents an innovative, evidence-based initiative that is the first of its kind in that it aims to increase access to depression screening for patients in primary care. Through the use of the iPad Depression Screening application, patients will be identified and referred to the Depression Educator. The DEI is designed to increase disease literacy, and dispel myths about depression and its treatment. A one-group pretest-posttest design will be used to asses knowledge of depression and its treatment and related stigma before, immediately after, and one month post education intervention.

#### Universal screening with iPad technology

The first step will involve training primary care providers and designated clinical staff in the use and interpretation of the PHQ-9 and the utilization of the iPad Depression Screening application. All adult primary care patients will be asked to complete the iPad Depression Screening (universal screening) at the annual visit and at new/non-acute visits. Using a brief script, medical assistants (MAs) will give the patient the iPad during the routine intake process (weight, blood pressure, etc.) and ask them to complete the screening for depression. Patients can refuse the depression screening process with no adverse effect to the services they receive at the clinic. The first screen in the iPad application will be used to record consent, and the patient will be informed that refusal to consent for screening will have no effect on the care they receive at the clinic. If the patient does not consent, the screening process will be terminated and the patient will continue with their scheduled visit.

The iPad Depression Screening application begins with the first two screening items of the PHQ-9 (the PHQ-2). The PHQ-2 is a validated “first-step” approach to screen for depression, which indicates if a patient should be further evaluated using the PHQ-9 [32]. For patients with scores above 3 on the first two items, the application automatically expands to the additional 7 items of the PHQ-9. The iPad Depression Screening will be presented in English or Spanish, depending on the patient’s preference. Once completed, an electronic notification is generated to the Depression Educator, the patient’s primary care provider and other designated clinic staff caring for the patient. Upon completion of the screening, the application screen will lock with a message to return the iPad to a clinic staff member.

**Fig. 1:**
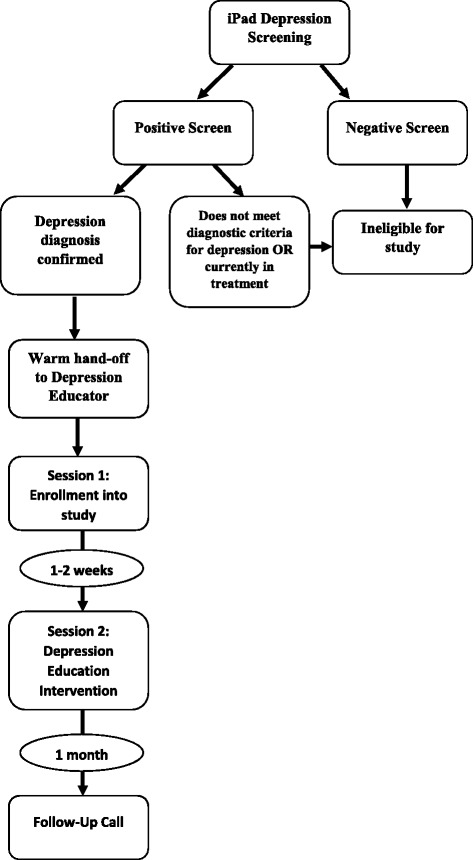
DESEO Participant Flow

Immediately after a patient screens positive for depression, the intake nurse will document the PHQ-9 score in the patient’s electronic health record (EHR). A notification will inform the provider that the patient screened positive for depression to enable the provider to interpret the depression score. Patients identified as depressed by the iPad Depression Screening application will be further assessed using the Major Depression Diagnostic Checklist (MDDC), a clinical interview tool used to confirm the nine criteria for depression from the Diagnostic and Statistical Manual of Mental Disorders (DSM-IV) [33]. The provider will then immediately facilitate an introduction to the Depression Educator, a practice known as a warm hand off, which establishes rapport and instills a sense of trust in the patient. Patients who do not meet the diagnostic criteria for depression will be excluded from the study. See Fig. 1 for a summary of the study flow.

#### Depression Education Intervention (DEI)

Patients identified as depressed by the iPad Depression Screening application (PHQ-9) will be referred to the Depression Educator for potential enrollment in the Depression Education Intervention (DEI). Patients who screen positive for depression but previously had a diagnosis of depression and are in treatment, will be excluded from enrollment in the DEI. The Depression Educator will confirm the diagnosis of major depression using the MDDC, if the provider has not already done so, and that the patient is not currently in treatment for depression or other mood disorder. The Depression Educator will explain the current project, including the compensation for completion of study measures at two sessions: baseline session to consent and enroll, and after completion of the DEI within 2 weeks. The Depression Educator will also explain that there will be one follow-up phone call one month after the completion of the DEI session. The Depression Educator will attempt to build rapport, engage the patient, and invite the patient to participate. If the patient agrees to participate in the DEI, s/he will be guided through the informed consent process. If a patient declines participation, s/he will receive routine care for their depression from the clinic providers.

The Depression Educator will document the patient’s PHQ-9 baseline score and administer the rest of the baseline measures, which includes the Depression Knowledge Measure (DKM) and three stigma measures (see Table 1). All measures will be documented in an electronic, password-protected research database. The Depression Educator will then schedule the patient to return to the clinic for the DEI. The patient will be encouraged to bring family members and loved ones to the session. The patient should be scheduled to return for the DEI within several days, at most within 2 weeks, of the first session.

**Table 1:** DESEO study assessments and frequency of administration

Assessment	Purpose	Screening	Baseline	2^nd^ Session (pre or post DEI)	Follow-Up
Patient Health Questionnaire (PHQ-9)	Measure depressive symptoms	X		pre	X
Depression Knowledge Measure (DKM)	Evaluate symptom recognition and treatment knowledge		X	post	X
Stigma Concerns About Mental Health Care (SCMHC)	Assess stigma related barriers to depression treatment utilization		X	post	X
Latino Scale for Antidepressant Stigma (LSAS)	Assess stigma related to the use of antidepressants		X	post	X
Social Distance (SD)	Measures desire for social distance from someone with depression		X	post	X

When the patient returns for the DEI, the Depression Educator will administer the PHQ-9 via the iPad Screening Application prior to the education intervention. The DEI uses a unique, culturally adapted depression *fotonovela* titled “Secret Feelings” developed by Cabassa, Molina and Baron [34]. The *fotonovela* is a popular comic-book style pamphlet that portrays a dramatic story using photographs and dialogue bubbles, which has become an effective tool for engaging Hispanic audiences and increasing knowledge about specific health issues [35, 36]. Using the *fotonovela* as a tool to promote discussion, the DEI is designed to enhance the awareness and understanding of depression, its role in chronic disease, its impact on Hispanic populations and the multitude of barriers to effective treatment. The DEI will be provided in one individual session by the Depression Educator in the presence of support by family members or loved ones, if desired. The DEI will be provided in English or Spanish depending on patient preference, to reduce stigma and enhance the comfort of patients to ask questions and discuss fears. Upon completion of the DEI, the patient will be asked to complete the DKM and the three stigma measures again. The Depression Educator will record the results of the measures and the second PHQ-9 in the research database. See Fig. 1 for study flow, Table 1 for summary of measures.

To determine the effectiveness of the intervention in engaging participant’s in depression treatment, the Depression Educator will call the patient one month after the DEI. The Depression Educator will assess if the patient is currently engaged in depression treatment of any kind (pharmacotherapy, counseling or other behavioral intervention). When possible, the information will be verified with the EHR. The Depression Educator will also administer all measures including the PHQ-9, DKM and three stigma measures (see Table 1).

### Measures

#### Patient Health Questionnaire (PHQ-9)

The PHQ-9 is a self-report of frequency of symptoms for “the last 2 weeks” on each of the nine Diagnostic and Statistical Manual of Mental Disorders (DSM-IV) [33] criteria for depression, which results in a range of possible scores from 0 to 27. PHQ-9 scores of 5 – 9 represent mild depression, 10 – 14 represent moderate depression, 15 – 19 represent moderately severe depression, and >20 represent severe depression [37]. Patients with a PHQ-9 score ≥10 are considered to have clinically significant depressive symptoms. Studies in primary care samples indicate the PHQ-9 to be a reliable and valid measure of depression severity with a Cronbach’s alpha of 0.89, and has demonstrated construct validity among African American, Latino and non-Hispanic white populations [38, 39]. The PHQ-9 has been translated and validated in Spanish. The screening is designed to provide requisite information while not overburdening patients and providers. The PHQ-9 will be administered at screening prior to enrollment in the DEI, at the second session before the DEI is conducted, and one-month post-DEI. See Table 1.

#### Depression Knowledge Measure (DKM)

The DKM [40] evaluates symptom recognition and treatment knowledge. Symptom recognition is assessed with a list of 10 symptoms, including 5 DSM-IV depression symptoms and 5 non-depressive symptoms: hearing voices, sleeping too little, eating too much, being full of energy, feeling guilty, feeling agitated, being violent, loss of interest, having hallucinations, and feeling confident. Respondents receive one point for each symptom that they identify correctly as a depression symptom or not a depression symptom. Treatment knowledge is assessed with 7 true–false questions from the Griffiths et al. [41] Depression Literacy Questionnaire (D-Lit) measure, adapted for use with Hispanic populations. Respondents receive one point for each question answered correctly. With 10 symptom recognition items and 7 treatment knowledge items, the DKM scores range from 0 (all incorrect) to 17 (all correct). The measure will be administered at baseline, after the Depression Education Intervention (DEI), and one-month post-DEI session. See Table 1.

Three brief stigma measures with demonstrated validity and reliability among Spanish-speaking, Hispanic primary care patients will be used to measure key stigma constructs which deter treatment utilization [42]. The three scales will be administered at baseline, after the DEI, and one-month post-DEI, see Table 1.

#### Stigma Concerns about Mental Health Care (SCMHC)

The SCMHC [42] is a 3-item measure which assess stigma related barriers to depression treatment utilization (sample item: “I would not want to receive treatment for depression because of being embarrassed to talk about personal matters with others”). Response options are scored as 0 = “Disagree” and 1 = “Agree.” The SCMHC score is calculated by taking the sum of the 3 items and total score ranges from 0 – 3. The SCMHC has demonstrated internal consistency with a Cronbach’s alpha of .84.

#### Latino Scale for Antidepressant Stigma (LSAS)

The LSAS [42] is a 7-item measure with stigma-related statements pertaining to use of antidepressants (sample item: “Prescription medicines for depression are for people who are not strong”). Respondents indicate whether they agree with the statements on a 3-point scale ranging from 0 = “No one thinks that way” to 2 = “Everyone thinks that way.” The LSAS score is calculated by taking the sum of the 7 items and total scores range from 0–14. The LSAS has demonstrated internal consistency with a Cronbach’s alpha of .80.

#### Social Distance (SD) scale

The SD [42] is a 6-item scale, which measures desire for social distance from someone with depression or history of depression treatment (sample item: “Would you socially interact with a person who is or had been in treatment for depression if this person moves next door?”). Respondents answer no, maybe, or yes to each item, scored as 0, 1, and 2, respectively. The SD score is calculated by taking the sum of items 1 – 6. Total score ranges from 0 to 12. Lower scores indicate greater social distance. The SD has demonstrated internal consistency with a Cronbach’s alpha of .75.

### Data monitoring and analysis

The data monitoring will focus on the proper collection of the depression measures, the depression knowledge measure, the stigma measures and patient outcomes after the DEI. A Project Coordinator will be responsible for monitoring the database and provide general support to the Depression Educator concerning issues related to DESEO. Such issues might include identifying problems with implementation, support to clinical staff to insure proper use of the iPad Depression Screening and Monitoring application, and facilitate the proper recording of scores in the EHR. Other duties include maintenance and oversight of the research database, tracking of supplies of depression education materials, and ensure continued fidelity of all clinic staff to the study protocols.

A sample size of 350 would be sufficient to find a statistically significant effect size of d = .176 (α = .05, Power [1-β] = .95). While the study is expected to produce a far greater effect size, a large sample size will provide additional analytic and investigative opportunities surrounding the psychometric properties and validity of the measures. The stigma measures, in particular, have not been tested in a primary care population and are of interest in identifying barriers to mental health treatment for Hispanic populations.

The primary analysis will assess changes in knowledge of depression pre-DEI, post-DEI and one month post-DEI using the Depression Knowledge Measure and changes in cultural attitudes toward depression pre and post DEI using the stigma measures (Table 1). Additional analyses will determine if the DEI leads to engagement in depression treatment for Hispanic patients by quantifying the number of Hispanic patients diagnosed with depression who went on to begin depression treatment after the intervention. Further analysis will quantify which treatment modality the patient with depression chose after the DEI: pharmacotherapy, counseling, or other behavioral intervention based on follow-up phone call one month post intervention and/or information in the EHR.

In order to evaluate the secondary aim of feasibility of universal screening for depression utilizing the PHQ-9 [31] via the iPad Depression Screening application, a qualitative analysis will determine if the providers and other clinic staff are introducing the screening with practiced scripts and conducting warm handoffs to the Depression Educator after screening positive for depression. Additional analyses will quantify via the iPad and clinic records if all adult patients are being screened and the score is being placed in the EHR.

Other analyses of interest would determine if the iPad Depression Screening application results in changes in primary care provider approach to the diagnosis and treatment of depression. Such analyses would include comparing the number of Hispanic patients with new a depression diagnosis as a percentage of the total number of adult Hispanics patients seen in the clinic compared to a matched set the year prior to the study based on the EHR (baseline data). Additionally, quantifying changes in the number of patients with a depression diagnosis being treated with antidepressants by a primary care provider as a percentage of the total number of Hispanics patients seen in the clinic compared to a matched set the year prior to the study based on the EHR (baseline data).

## Discussion

This study is unique in that it aims to examine the specific role of patient education as an intervention to increase engagement in depression treatment, as opposed to studies which provide patient education as a component of a larger depression treatment intervention. It is expected that the DEI will reduce barriers to treatment of depression for Hispanic patients by increasing knowledge of the disorder (disease literacy), its causes, symptoms and its role in chronic disease. Discussion of myths related to treatment will help allay fears of engaging in treatment. By participating in the DEI, it is expected that patients will have time to understand treatment options, participate in shared decision making with their provider about treatment, and increase engagement in treatment of depression which will lead to improved overall health.

While it is expected that implementation of the iPad Depression Screening application will increase identification of a large number of patients whose depression was previously unrecognized and might increase the workload of the primary care providers, with the support of the DEI, the burden of patient education about the disease and its treatment options will be reduced. Additionally, the recognition and treatment of depression will improve the treatment of comorbid chronic disease and ultimately reduce the burden on the primary care practice.

## Abbreviations

CMS, Center for Medicare and Medicaid Services; DEI, Depression Education Intervention; DESEO, Depression Screening and Education: Options to Reduce Barriers to Treatment; DKM, Depression Knowledge Measure; D-Lit, Depression Literacy questionnaire; DSM-IV, Diagnostic and Statistical Manual of Mental Disorders; EHR, electronic health record; LSAS, Latino Scale for Antidepressant Stigma; MDDC, major depression diagnostic checklist; PHQ-2, first two screening items of the PHQ-9; PHQ-9, 9-item Patient Health Questionnaire; SCMHC, Stigma Concerns about Mental Health Care; SD, Social Distance scale
